# Resilience measurement scale in family caregivers of children with cancer: Multidimensional item response theory modeling

**DOI:** 10.3389/fpsyt.2022.985456

**Published:** 2023-01-16

**Authors:** Said Jiménez, José Moral de la Rubia, Rosa María Varela-Garay, Cesar Merino-Soto, Filiberto Toledano-Toledano

**Affiliations:** ^1^Unidad de Investigación en Medicina Basada en Evidencias, Hospital Infantil de México Federico Gómez, National Institute of Health, Mexico City, Mexico; ^2^Facultad de Psicología, Universidad Autónoma de Nuevo León, Monterrey, Mexico; ^3^Departamento de Trabajo Social y Servicios Sociales, Facultad de Ciencias Sociales, Universidad Pablo de Olavide, Seville, Spain; ^4^Instituto de Investigación en Psicología, Universidad de San Martin de Porres, Lima, Peru; ^5^Unidad de Investigación Sociomédica, Instituto Nacional de Rehabilitación Luis Guillermo Ibarra Ibarra, Mexico City, Mexico; ^6^Dirección de Investigación y Diseminación del Conocimiento, Instituto Nacional de Ciencias e Innovación para la Formación de Comunidad Científica, INDEHUS, Mexico City, Mexico

**Keywords:** resilience, psychometric properties, family caregivers, cancer, item response theory

## Abstract

**Background:**

Currently, information about the psychometric properties of the Resilience Measurement Scale (RESI-M) in family caregivers of children with cancer according to item response theory (IRT) is not available; this information could complement and confirm the findings available from classical test theory (CTT). The objective of this study was to test the five-factor structure of the RESI-M using a full information confirmatory multidimensional IRT graded response model and to estimate the multidimensional item-level parameters of discrimination (MDISC) and difficulty (MDIFF) from the RESI-M scale to investigate its construct validity and level of measurement error.

**Methods:**

An observational study was carried out, which included a sample of 633 primary caregivers of children with cancer, who were recruited through nonprobabilistic sampling. The caregivers responded to a battery of tests that included a sociodemographic variables questionnaire, the RESI-M, and measures of depression, quality of life, anxiety, and caregiver burden to explore convergent and divergent validity.

**Results:**

The main findings confirmed a five-factor structure of the RESI-M scale, with RMSEA = 0.078 (95% CI: 0.075, 0.080), TLI = 0.90, and CFI = 0.91. The estimation of the MDISC and MDIFF parameters indicated different values for each item, showing that all the items contribute differentially to the measurement of the dimensions of resilience.

**Conclusion:**

That regardless of the measurement approach (IRT or CTT), the five-factor model of the RESI-M is valid at the theoretical, empirical, and methodological levels.

## 1. Introduction

Childhood cancer has serious repercussions on the physical and psychological health of pediatric patients, their families and their caregivers; caregiving can be experienced as a stressful process that can cause psychological and physical effects and consequences (1–4). Childhood cancer patients and their families often experience anxiety, depression, and parental stress (5–7); poor health (8); and social and economic overload and caregiver burnout (9). Therefore, caregiving has effects on the quality of life, caregiver profile and resilience of families caring for children with cancer (10, 11). The research literature has identified a number of contextual factors and sociodemographic characteristics in family caregivers of pediatric patients that increase the risk for physical and psychological health impacts (12). The main demographic variables include gender (13), unemployment (5), low income (14), low levels of education (15), social support networks (16), caregiver marital status (17), number of children in the family (18), child age (19), and the psychosocial profile of family caregivers (11). Contextual factors include the time elapsed since diagnosis (20), subjective perceptions of disease severity of both patients and caregivers (21), the duration of the disease (22), the personality type of the parents (23), and the duration and impact of care (24, 25). In this regard, evidence indicates that the term “caregiver”, which was first used in 1966 referring to those “helping those who suffer”, is a multidimensional construct, and its use in research lacks a coherent conceptualization and an operational definition (26). However, in chronic illness contexts, the family caregiver has been defined as the person who has a significant emotional bond with the patient; who may be a family member who is part of the patient’s family life cycle; who offers emotional, expressive, instrumental and tangible support; and who provides assistance and comprehensive care during the chronic illness, acute illness or disability of a child, adult, or elderly person (11). In this sense, resilience to chronic illness is a process of positive adaptation despite the loss of health, which implies the development of vitality and skills to overcome the negative effects of adversity, risk, and vulnerability caused by the disease (27).

The measurement and assessment of resilience depends on how it is defined, and the factors associated with it (28). One of the measurements developed for Mexican population was the Resilience Measurement Scale (RESI-M) (29). It is an instrument of 43 items with a Likert-type scale with four response options, ranging from 1 “totally disagree” to 4 “totally agree.” The items of the RESI-M were derived from two instruments that measure resilience that are widely used in the international literature, namely, the Connor-Davidson Resilience Scale (CD-RISC) (30) and the Resilience Scale for Adults (RSA) (31). Both scales measure resilience in adults. According to Palomar and Gómez (29), the factors of the RESI-M have been defined as follows: (1). Strength and Self-Confidence refers to the clarity that individuals have about their objectives, the effort they make to achieve their goals, the confidence they have that they will succeed and the optimism, strength and tenacity with which they face their challenges. (2). Social Competence indicates the competence of individuals to relate to others and the ease with which they make new friends, make people laugh and enjoy a conversation. (3). Family Support addresses family relationships and family support, loyalty among family members, and family members sharing similar views of life and spending time together. (4). Social Support, mainly from friends, points to the individual having people who can help, give encouragement and care about him or her in difficult times. (5). Structure refers to the ability of people to organize themselves, to plan activities and time, and to have rules and systemic activities, even in difficult times.

The research literature on the psychometric properties of the RESI-M in different contexts and Mexican subpopulations has shown empirical evidence that it is a valid and reliable scale. In this regard, in a sample of 348 Mexican adults (235 women and 113 men), the psychometric properties of the RESI-M were evaluated, the structure was reproduced by means of principal component analysis, and 58.71% of the variance explained by the five factors was reported, the overall internal consistency was high (α = 0.92) (32). In another study conducted by Sanjuan-Meza et al. (33) with indigenous women in Mexico, the results of the psychometric analysis of the RESI-M showed a final version of the instrument with 34 questions (out of the original 43), acceptable reliability (α = 0.942), and six factors that explained 56.34% of the total variance (33). In another validation study of the RESI-M in patients with chronic renal failure treated with hemodialysis, after exploratory factor analysis, two of the 43 items were eliminated. The five factors explained 63.6% of the total variance, with an overall α = 0.96, and the five factors were negatively correlated with symptoms of anxiety, depression, and distorted thoughts (34). Another study aimed to obtain the psychometric properties of the RESI-M in family caregivers of children with chronic conditions (35) and showed an adequate fit with the data based on a maximum likelihood estimator. The overall internal consistency was 0.95, and the variance explained was 63%. Likewise, in a validation study of the RESI-M in family caregivers of children with cancer, the RESI-M showed reliability and construct validity and overall internal consistency (α = 0.976), and the explained variance was 47%. Confirmatory factor analysis showed that the five-factor model fit the data well: NFI = 0.970, CFI = 0.997, SRMR = 0.055, and RMSEA = 0.019. The RESI-M scale total score was positively correlated with psychological well-being and negatively correlated with depression, parental stress, and anxiety (27).

The findings obtained in these studies suggest that (a) the RESI-M is a multidimensional measure representing psychosocial and individual aspects of resilience; (b) the dimensions of the RESI-M remain stable; (c) the dimensions of the RESI-M are correlated, such that they would covary in the resilient behavior exhibited by the individual in situations in general; (d) the covariation of these attributes in behavior is not, however, equal among the dimensions, to the degree that some would covary more strongly than others; (e) the content of the construct of resilience appears to be unstable across studies because the number of items does not remain the same across studies (i.e., a small number, and different items need to be eliminated); and (f) the methods for studying internal structure have used an approach based on linear models.

Research regarding the impact of resilience on family caregivers is promising, but one of its limitations is having reliable measurement instruments that have been validated in this specific population. The RESI-M can be useful for this purpose and has the advantage of having been developed in the international cultural context. However, it should be considered that this test was originally validated for use in the general population; therefore, its use in other specific populations, such as family caregivers of children with cancer, would compromise the validity and reliability of its results due to the lack of psychometric data. Although there is scientific evidence in the literature about the validity and reliability of the RESI-M in various Mexican contexts, no research results have been found that show empirical findings of the psychometric properties of RESI-M having been analyzed, evaluated and studied based on the item response theory (IRT) in a population of family caregivers of children with cancer. The IRT framework takes into account the non-linear relationship of the items with the latent attribute and the categorical expression of the items to represent the participants’ responses to the measurement instrument (36). One of the main advantages of item calibration in the IRT framework is the psychometric properties provided by graded response modeling (37). In this model, the importance of each item in the measurement of the construct it is intended to measure is weighted, as opposed to classical test theory (CTT), which assumes that all items contribute equally to the measurement of the construct. Another advantage of the analysis in the IRT framework is in terms of the reliability of the instrument since the information functions allow the exploration of the accuracy of the measurements of the RESI-M factors depending on a range of values in the constructs. In contrast, CTT assumes that measurement reliability is the same at all levels of measured traits (36). Within this IRT framework, as one of the models applied to polytomous items (i.e., ordinal or Likert responses), the graded response model (GRM) has gained much acceptance because it models the variability of item discrimination and threshold spacing (36, 37), which is more realistic for most psychosocial measures.

In response to this need for reliable measurement instruments of resilience for the family caregiver population as well as to the existing knowledge gaps and to bridge the gap in this field of knowledge, the aim of the present study was to analyze the psychometric properties of the RESI-M. To this end, we formulated six objectives: (1). To evaluate the five-factor structure of the RESI-M using a full-information confirmatory and multidimensional IRT GRM; (2). To estimate the multidimensional item-level parameters of discrimination (MDISC) and difficulty (MDIFF) from the RESI-M scale; (3). To plot the item characteristic curves (ICCs) of the RESI-M; (4). To calculate the estimated precision of latent traits using the information functions of the five factors of the RESI-M; (5). To obtain measurements of the five latent factors of the RESI-M for cancer patients’ caregivers; and (6). to investigate test score validity by correlating the measurements of the five latent factors with the total scores of the Beck Depression Inventory (BDI) (38), Beck Anxiety Inventory (BAI) (39), WHOQoL-BREF (40), and Zarit Burden Interview (ZBI) (41). Taking the antecedent validation studies of the RESI-M as a framework, hypothesis regarding the psychometric content were formulated. In relation to dimensionality, the hypothesis was that the number of dimensions of the RESI-M would remain at five dimensions; the second hypothesis was that the dimensions of the RESI-M would be correlated. The third hypothesis was that the items would show high levels of discrimination. Regarding relationships with external variables, a negative linear association was expected with maladaptive responses, such as anxiety symptoms, depression symptoms, and subjective burden symptoms, and a positive linear association was expected with adaptive responses, such as quality of life.

## 2. Materials and methods

### 2.1. Participants

A non-experimental, transversal, *ex post facto* study was conducted using a convenience and non-probabilistic sampling technique. A total of 633 family caregivers of hospitalized children with cancer were interviewed at the Hospital Infantil de Meìxico Federico Goìmez National Institute of Health in Mexico City. The sample included women (81.4%) and men (18.6%) aged between 18 and 52 years, with an average of 31.7 years (SD = 7.6). The inclusion criteria for the study were (1) being a family caregiver of a child who was receiving cancer treatment, (2) being at least 18 years old, and (3) having signed an informed consent form. The exclusion criteria were (1) inability to read and write and (2) refusal to participate in the study. The deletion criteria included partial or incomplete responses to the psychosocial measurement instruments. The pediatric patients included both girls (47.7%) and boys (52.3%) aged between 1 and 17 years, with an average age of 5.8 (SD = 4.9). In most cases, the time elapsed since cancer diagnosis ranged from one week to one year (68.4%), and the hospitalization period was one week to one month (85.3%).

### 2.2. Instruments

A battery of test instruments, including a sociodemographic variables questionnaire for research with families of children with chronic diseases and four self-report instruments measuring psychosocial variables (resilience, depression, anxiety, quality of life, and caregiver burden), were used. To guarantee the accuracy of the data obtained, the instruments were validated in the Mexican population and with families of children with chronic diseases.

#### 2.2.1. Sociodemographic variables questionnaire (Q-SV) for research with family caregivers of children with chronic diseases

This questionnaire contains 20 items that evaluate information on sociodemographic, medical, sociocultural and family variables in families of children with chronic diseases. For this study, the diagnosis, the age and sex of the patient and caregiver, the relationship between the patient and caregiver (mother, father, or another family member), the educational level (no schooling, primary education, secondary education, undergraduate education, postgraduate education), occupation (homemaker, worker, trader, employee, student, pensioner, unemployed), marital status (married, living together, separated, divorced, single parent, widowed), years of partnership, number of children, type of family (nuclear, seminuclear, extended, single-parent), family life cycle (with young children, with school-age children, with adult children), social support networks (family, friends, religion, institutions, government), religion (Catholic, Christian, none), and monthly income were determined (12).

#### 2.2.2. Resilience measurement scale in Mexicans (RESI-M)

This scale has been validated in family caregivers of children with cancer (35). This scale contains 43 four-point Likert-type items, ranging from 1 “strongly disagree” to 4 “strongly agree,” and measures the level of overall resilience and five dimensions: Strength and Self-Confidence (19 items), Social Competence (eight items), Family Support (six items), Social Support (five items), and Structure (five items) (29).

#### 2.2.3. Beck depression inventory II (BDI-II)

This inventory has been validated in a population of family caregivers of children with chronic diseases (42). This inventory includes 21 items, each with four statements that assess depressive symptomatology and episodes. It uses a rating scale from 0 to 3, where the higher the score is, the higher the level of depression. The level of depression is interpreted as follows: minimum from 1 to 4, mild from 5 to 13, moderate from 14 to 27, and severe from 28 to 63 points. Among the 330 family caregivers in the present study, the overall internal consistency of the 21 items was excellent (α = 0.90; 95% CI = 0.89, 0.91; ω = 0.92; 95% CI = 0.91, 0.94) (38).

#### 2.2.4. Beck anxiety inventory (BAI)

This instrument has been validated in family caregivers of children with cancer by Toledano-Toledano et al. (43). With 16 items, this inventory assesses anxious symptomatology using a four-point scale, ranging from 0 “Little or nothing” to 3 “Severely.” The level of anxiety obtained is minimum (1 to 5 points), mild (6 to 15), moderate (16 to 30), or severe (31 to 63). In the present sample, the overall internal consistency of the 21 items was excellent (α = 0.94; 95% CI = 0.94, 0.95; ω = 0.97; 95% CI = 0.96, 0.98) (39).

#### 2.2.5. WHOQOL-BREF inventory of quality of life

This inventory has been validated in a Mexican population (40). It includes 26 five-point Likert-type items ranging from 1 to 5. Two items constitute general questions about quality of life, and the remaining items are grouped into the following dimensions: physical health (seven items), psychological health (six items), social relations (three items), and environment (eight items). Among the 330 family caregivers in the present study, the overall internal consistency of the 26 items was excellent (α = 0.92) (40).

#### 2.2.6. Zarit burden interview (ZBI)

This instrument has been validated in a Mexican population (44). It assesses the subjective burden, attitudes and emotional reactions of the caregiver when faced with the responsibility of care and the perception of the situation. It contains 22 items distributed across three factors: impact of caregiver (13 items), interpersonal relationship (six items), and self-efficacy expectations (three items). The scores of the items range from 0 “Never” to 4 “Always.” In the present study, only the ZBI total score was used, and its overall internal consistency was excellent among the 330 family caregivers (α = 0.85; 95% CI = 0.82, 0.87; ω = 0.98, 95% CI = 0.93, 1.00) (41).

### 2.3. Procedure

The family caregivers were interviewed by the corresponding author of this study in the wards of the Hematology-Oncology Service of the Hospital Infantil de México Federico Gómez, National Institute of Health. All the family caregivers interviewed were invited to participate voluntarily; the objectives of the research were explained to them, and all of their concerns regarding the study were addressed. The family caregivers who agreed to participate signed informed consent forms and answered the instruments individually during a single session. Participants did not face any consequences for withdrawing their consent, as specified on the informed consent sheet. Before collecting the completed instruments, the interviewer checked that there were no questions without answers. If there were questions without answers, the participant was asked to respond to them, and in this way, we managed to avoid missing values.

### 2.4. Ethical considerations

This study is a part of the research project HIM/2015/017/SSA.1207 “Effects of mindfulness training on psychological distress and quality of life of the family caregiver,” which was approved on December 16, 2014, by the Research, Ethics, and Biosafety Commissions of the Hospital Infantil de México Federico Gómez, National Institute of Health, in Mexico City. While conducting this study, the ethical rules and considerations for research with humans currently enforced in Mexico (45) and those outlined by the American Psychological Association (46) were followed. All family caregivers were informed of the objectives and scope of the research and their rights according to the Declaration of Helsinki (47). The caregivers who agreed to participate in the study signed an informed consent letter. Participation in this study was voluntary and did not involve payment.

### 2.5. Statistical analyses

#### 2.5.1. Item response theory modeling

A confirmatory multidimensional IRT model was used in which five correlated factors were a priori specified to evaluate the structure and psychometric properties of the RESI-M. To evaluate their robustness in comparison with alternative measurement models, competing models were also specified: unidimensional (representing the absence of differentiated content and scores), multidimensional orthogonal (including the specific factors but restricting the correlations between them) and bifactor (representing the coexistence of a general factor and specific factors). As the scale is composed of polytomous items with ordered response categories, the GRM (37) was used, and its parameters were estimated with the Metropolis-Hastings Robbins-Monroe (MHRM) method using the “mirt” package in R (48). To facilitate model interpretation, the GRM’s slopes and thresholds were re-parametrized according to Reckase (36) to obtain the multidimensional discrimination (MDISC) and difficulty (MDIFF) parameters. The goodness of fit of the models was evaluated using the *M*_2_ * statistic and its associated RMSEA value; other fit indices were also obtained (e.g., CFI > 0.95, SRMR < 0.05). In the evaluation of the bifactor model, the extracted common variance [ECV; (49)], which indicates the degree of common variability derived from the general factor, was additionally estimated. ECV > 0.70 suggests essential unidimensionality (50). Likewise, ICCs were calculated, and the information functions of the five factors in the RESI-M scale were calculated. The ICCs allowed the investigation of the response probabilities to each category across the range in the latent trait θ, while the information functions indicated the change in the precision of the estimates in a range of −4≤θ≤4. Finally, the measurements in the 5 factors of the 633 caregivers were obtained, and for the sake of validity, their linear relation with total scores of the BDI, the BAI, the WHOQoL-BREF, and the ZBI was computed using simple linear regression controlling by sex and age of the caregiver.

#### 2.5.2. Linear model

For comparability with previous RESI-M studies, the linear model was used to estimate the internal consistency coefficients α and ω, with confidence intervals (95%) generated by bootstrap sampling (*n* = 1,000 samples). This procedure was implemented by the *omega* command (51).

## 3. Results

### 3.1. Characteristics of the family caregivers

The sample included 515 women (81.4%) and 116 men (18.6%) aged between 18 and 49 years, with an average age of 31.6 (SD = 7.5). Regarding education, 2.7% of the participants had no education, 19.7% had primary school education, 44.6% had secondary school education, 25.5% had upper secondary (high school) education, and 7.4% had university or college education. The median and mode of the number of children was two, ranging from 0 to 10. More details are provided in Table 1. The pediatric patients included both girls (47.7%) and boys (52.3%), aged between 1 and 17 years, with an average age of 5.8 (SD = 4.9). In most cases, the time elapsed since cancer diagnosis ranged from one week to one year (68.4%), and the hospitalization period was one week to one month (85.4%).

**TABLE 1:** Summary statistics of sociodemographic variables.

Sociodemographic variable		*N*	%
**Sex**			
	Men	118	18.6
	Women	515	81.4
**Schooling**			
	No schooling	18	2.8
	Primary	124	19.6
	Secondary	282	44.5
	Higher secondary (high school)	163	25.8
	University or college	46	7.3
**Occupation**			
	Homemaker	413	65.2
	White-collar worker	87	13.7
	Merchant	58	9.2
	Blue-collar worker	26	4.1
	Unemployed	49	7.7
**Marital status**			
	Married	257	40.6
	Living together	244	38.5
	Separated	53	8.4
	Single mother	53	8.4
	Divorced	18	2.9
	Widowed	6	0.9
	Other	2	0.3
**Income per month**			
	<141 US dollars	390	61.6
	Between 141 and 281 US dollars	140	22.1
	Between 282 and 563 US dollars	85	13.4
	>563 US dollars	18	2.8
**Religious adscription**			
	Catholic Christian	512	80.9
	Non-Catholic Christian	75	11.8
	No religion	46	7.3
			
		M	SD
Age (years)		31.7	7.58
Number of children		2.32	1.17

*n*, frequency; %, percentage; mean, arithmetic mean; SD, standard deviation.

### 3.2. Model results

#### 3.2.1. Internal structure and model fit

From all competing models, the multidimensional IRT model with correlated factors and the bifactor model obtained the best goodness of fit indices (Table 2). The bifactor model yielded lower RMSEA and higher TLI and CFI values than the multidimensional IRT model with correlated factors; however, the ECV derived from the primary factor was 0.62, which weakens the conclusion that a bifactor structure underlies the RESI-M (50) and was the reason why we decided to report on the functioning of the multidimensional IRT model with correlated factors. Even though this confirmatory model had a statistically significant value of *M*_2_*(774) = 3714.12, *p < .001*, the RMSEA suggested an acceptable fit, with *RMSEA* = 0.078 (95% CI: 0.075, 0.080), as did the TLI and CFI statistics, which were 0.90 and 0.91, respectively.

**TABLE 2:** Goodness-of fit-indices and information criteria from all competing models.

Model	ML	df	*p*	RMSEA	TLI	CFI	AIC	BIC	loglik
Unidimensional	7199.27	774	<0.001	0.12	0.78	0.80	43,388	44,154	−21,522
MD (orthogonal)	3718.36	774	<0.001	0.08	0.90	0.91	41,084	41,849	−20,370
MD (correlated factors)	3714.12	774	<0.001	0.08	0.90	0.91	39,816	40,626	−19,726
Bifactor	2587.33	731	<0.001	0.06	0.93	0.94	39,529	40,486	−19,549

MD, multidimensional; ML, likelihood-ratio-chi-2 test statistics; df, degree of freedom; *p*, probability value; AIC, Akaike information criterion; RMSEA, root mean square error of approximation; TLI, Tucker–Lewis index; BIC, Bayesian information criterion; loglik, log likelihood.

#### 3.2.2. Multidimensional item parameters

The multidimensional parameters (MDISC and MDIFF) of the RESI-M obtained in the sample of caregivers are included in Table 3. In IRT, the *a* parameter corresponds to the slope of the function, in this case, MDISC (36), which allows individuals with low or high levels of the latent trait to be distinguished. Likewise, the parameters *b*_1_, *b_2_*, and *b_3_* that correspond to the thresholds are presented as measurements of MDIFF (36), which indicates how much of the latent trait is required for a respondent to endorse a particular category. Items with a greater_*a*_ value have better discrimination (i.e., they have a stronger relationship with the latent construct), and response categories with a larger *b* value indicate that the caregiver must have a high level of *resilience* to select that category. The range of *a* values was from 1.40 for item 2 of the Strength and Self-Confidence factor to 4.88 for item 35 of the Social Support factor; therefore, according to the classification proposed by Baker (52), 19% of the items had “high” discrimination, while the majority (81%) had “very high” discrimination.

**TABLE 3:** Multidimensional parameters of discrimination and difficulty from the full information confirmatory graded response model (GRM).

Factor and item		a	b_1_	b_2_	b_3_
**Strength and Self Confidence (SSC)**					
1	What has happened to me in the past makes me feel confident…	1.50	−2.77	−1.62	0.69
2	I know where to look for help.	1.41	−3.00	−1.64	0.98
3	I am a strong person.	1.94	−3.31	−1.67	0.64
4	I know very well what I want.	2.25	−3.14	−1.48	0.62
5	I have control over my life.	1.57	−3.01	−1.37	1.14
6	I like challenges.	1.51	−2.84	−1.26	1.05
7	I strive to reach my goals.	3.29	−2.73	−1.89	0.34
8	I am proud of my achievements.	2.96	−2.67	−1.56	0.39
9	I know I have skills.	3.24	−2.61	−1.97	0.25
10	Believing in myself helps me overcome difficult moments.	2.27	−2.99	−1.89	0.20
11	I think I will succeed.	2.37	−2.76	−1.68	0.38
12	I know how to achieve my goals.	3.03	−2.98	−1.28	0.73
13	Whatever happens, I will always find a solution.	2.08	−3.14	−2.08	0.39
14	My future looks good.	2.53	−2.51	−1.01	0.90
15	I know that I can solve my personal problems.	2.78	−3.35	−1.96	0.53
16	I am satisfied with myself.	2.86	−2.59	−1.46	0.62
17	I have realistic plans for the future.	1.96	−2.86	−1.62	0.71
18	I trust my decisions.	2.70	−3.36	−1.53	0.62
19	When I am not well, I know that better times will come.	1.62	−3.32	−2.39	0.44
**Social Competence (SC)**					
20	I feel comfortable with other people.	1.60	−2.79	−1.19	1.32
21	It is easy for me to establish contact with new people.	1.96	−2.32	−0.93	1.28
22	It is easy for me to make new friends.	2.20	−2.15	−0.77	1.18
23	It is easy for me to think of good topics of conversation.	2.96	−2.32	−0.80	0.99
24	I adapt easily to new situations.	2.20	−2.33	−0.93	1.06
25	It is easy for me to make other people laugh.	1.67	−2.80	−0.57	1.66
26	I enjoy being with other people.	1.84	−3.10	−1.29	1.35
27	I know how to start a conversation.	2.52	−2.50	−0.92	1.20
**Family Support (FS)**					
28	I have a good relationship with my family.	3.20	−2.27	−1.62	0.14
29	I enjoy being with my family.	3.56	−2.87	−1.86	−0.19
30	In our family, we are loyal to each other.	4.48	−2.13	−1.48	0.18
31	In our family, we enjoy doing activities together.	4.81	−2.09	−1.35	0.07
32	Even in difficult times, our family has an optimistic attitude…	1.94	−2.45	−1.86	0.46
33	In our family we agree in relation to what we consider…	1.90	−3.34	−1.99	0.53
**Social Support (SS)**					
34	I have some friends/relatives who truly care about me.	3.99	−1.95	−1.43	0.30
35	I have some friends/relatives who support me.	4.88	−1.86	−1.27	0.34
36	I always have someone who can help me when I need it.	3.15	−1.97	−1.37	0.28
37	I have some friends/relatives who encourage me.	4.35	−2.02	−1.36	0.30
38	I have some friends/relatives who value my skills.	3.14	−2.12	−1.37	0.64
**Structure (Str)**					
39	Rules and routine make my life easier.	1.83	−2.42	−0.89	1.45
40	I keep my routine even in difficult times.	1.59	−2.51	−0.62	1.74
41	I prefer to plan my activities.	1.94	−2.39	−1.11	1.41
42	I work better when I have goals.	1.81	−3.07	−1.68	0.94
43	I am good at organizing my time.	2.30	−2.11	−0.81	1.16

a: slope parameter (discrimination). b_*i*_: thresholds parameter.

#### 3.2.3. Information functions

**FIGURE 1:**
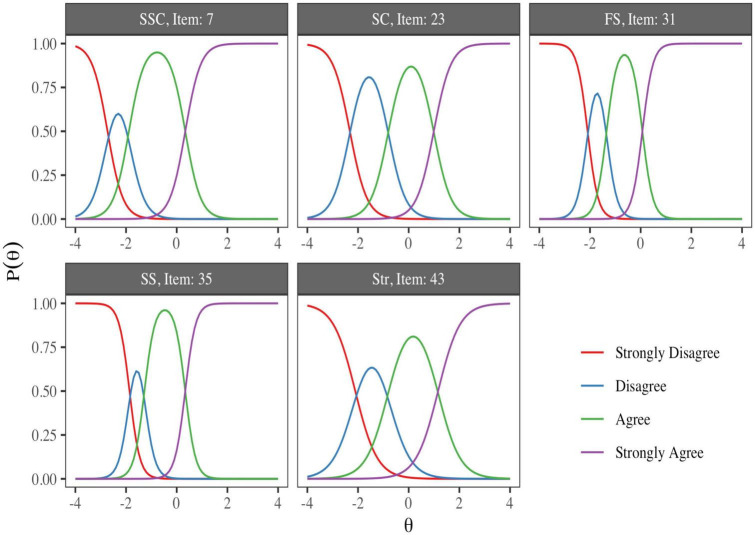
Item characteristic curves with the greatest discrimination from each factor of the resilience measurement scale (RESI-M) scale. The ICCs reveal the GRM response predictions at different levels of the Strength and Self-Confidence (SSC), Social Competence (SC), Family Support (FS), Social Support (SS), and Structure (Str) factors.

Figure 1 shows the ICCs of the items with the highest discrimination of each of the five factors. Each panel includes the probability of selecting the response categories depending on a range of −4≤θ≤4 in the latent trait. The ICCs reveal the GRM response predictions across different levels of the Strength and Self-Confidence (SSC), Social Competence (SC), Family Support (FS), Social Support (SS), and Structure (Str) factors. As the scores in the latent trait are standardized, the average of the scale occurs when θ = 0; at this level of the traits, it is possible to observe that the most likely response to these items is the category “Agree.” Levels above the average of the latent traits are required to select the highest response category, and levels below θ < −1 are required to select the lowest categories.

#### 3.2.4. Score reliability

**FIGURE 2:**
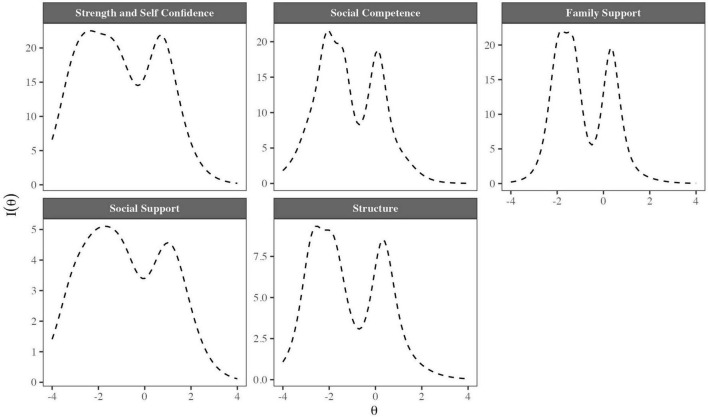
Test information functions (TIFs) for the five factors of the RESI-M.

Additionally, the test information functions (TIFs) for the five RESI-M factors are shown in Figure 2. The TIFs allow the test precision to be explored to measure different levels of the traits. At the levels of θ where the function increases, we found the most precise measures; this is also where the test can collect the most information from the latent traits. For example, the Strength and Self-Confidence factor TIF provided information in a wide range of θ values; however, the information was substantially higher for values lower than the average when θ≈−2. Additionally, it could be observed that the function had another maximum at levels above the average (θ≈1), and that pattern was present in all factor functions.

In the linear modeling, α coefficients for the RESI-M scores were as follows: *Strength and Self-Confidence* (α = 0.93, 95% CI = 0.92,.94, se = 0.004), Social Competence (α = 0.86, 95% CI = 0.83, 0.88, se = 0.011), Family Support (α = 0.88, 95% CI = 0.87,.90, se = 0.009), Social Support (α = 0.91, 95% CI = 0.89, 0.93, se = 0.008), and Structure (α = 0.75, 95% CI = 0.71, 0.79, se = 0.019). Additionally, the ω coefficients for the factors were as follows: Strength and Self Confidence (ω = 0.95, 95% CI = 0.94, 0.96), Social Competence (ω = 0.88, 95% CI = 0.86, 0.90), Family Support (ω = 0.94, 95% CI = 0.91, 0.96), Social Support (ω = 0.92, 95% CI = 0.90, 0.94), and Structure (ω = 0.78, 95% CI = 0.74, 0.81).

#### 3.2.5. Factors’ individual scores

**FIGURE 3:**
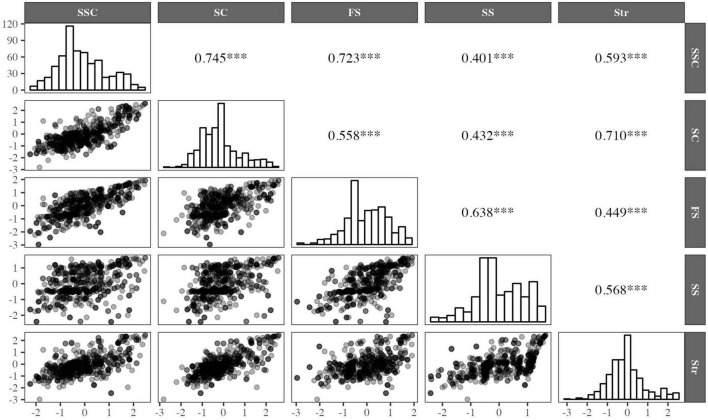
Estimation of individual factor scores, their dispersion and Pearson’s correlation coefficient. SSC, Strength and Self-Confidence; SC, Social Competence; FS, Family Support; SS, Social Support; Str, Structure. ^***^p < 0.001.

The estimation of the factors’ individual scores, their dispersion, and Pearson’s correlation coefficient are shown in Figure 3. The distributions of the standardized scores of the factors are included in the figure diagonal. In the lower part of the matrix, the figures depict the position of each caregiver in two dimensions as points of the scatter plots, while in the upper part of the matrix, the Pearson correlation coefficients are reported, which were positive and statistically significant (*p* < .001). A slight positive bias can be observed in the distributions of the Strength and Self-Confidence (SSC), Social Competence (SC), and Structure (Str) factors, as well as a slight negative bias for the Family Support (FS), and Social Support (SS) factors. Therefore, it can be inferred that the majority of the caregivers had low scores on the Strength and Self-Confidence, Social Competence, and Structure factors, while the Family Support and Social Support scores of the majority of the caregivers were high. Regarding the correlations of the factors, positive and strong associations (*r* > .7) were detected for the relationships between the Strength and Self-Confidence and Social Competence factors, Strength and Self-Confidence and Family Support factors, and Structure and Social Competence factors.

#### 3.2.6. Validity

Finally, regarding the validity of the measures of latent traits in primary caregivers, the linear relation between the five factors of the RESI-M and the total scores of the BDI, BAI, WHOQoL-BREF, and ZBI provided evidence of convergent and discriminant validity. Table 4 includes the standardized slope parameters matrix (simple linear regression coefficients) between all the RESI-M factors and the total scores of the aforementioned scales controlling by sex and age of the caregiver. In general, it can be noted that the scores of the resilience factors predict negative relations with depression (BDI), anxiety (BAI), and caregiver burden (ZBI) and a positive association with the quality-of-life scale (WHOQoL-BREF). Although the strength of the relationships varied from weak to moderate estimates, all of them were statistically significant at *p* < .05.

**TABLE 4:** Standardized linear regression slopes to evaluate the validity of factor score measures in primary caregivers.

	Linear correlations				Descriptive information			
Variable	BDI	WHOQoL	BAI	ZBI	M	SD	Sk	K
SSC	−0.44	0.54	−0.23	−0.23	6.19	8.36	0.15	−0.47
SC	−0.34	0.46	−0.18	−0.20	22.98	3.97	0.36	0.13
FS	−0.45	0.53	−0.31	−0.23	19.91	3.25	−0.84	1.22
SS	−0.17	0.39	−0.13	−0.22	16.12	3.09	−0.84	1.39
Str	−0.24	0.34	−0.10	−0.17	14.43	2.44	0.19	0.78

SSC, Strength and Self Confidence; SC, Social Competence; FS, Family Support; SS, Social Support; Str, Structure; Sk, Fisher’s skew coefficient; K, Fisher’s excess kurtosis. Slopes were obtained controlling by sex and age of the caregiver. All *p*-values were statistically significant at *p* < 0.05.

## 4. Discussion

The aim of the study was to evaluate the construct validity of the RESI-M, focusing on the internal structure, the reliability of the scores, and the relationship with external constructs. In contrast to previous studies of the RESI-M (27, 35), the present study used a full-information confirmatory multidimensional IRT GRM, a model that allows parameters of the metric structure of the instruments to be obtained in a non-linear framework and that is more detailed at the item level and at the score level. The results of our evaluation support the multidimensional structure of the RESI-M. We confirmed the five dimensions of the scale proposed in previous evaluations conducted with a linear analysis framework. In terms of item functioning, all 43 items of the RESI-M were informative (i.e., the degree to which they contain information about the construct measured) and contributed specifically to assessing different aspects of resilience. Adequate item functioning comprised five latent dimensions that accurately measure the factors Strength and Self-Confidence (SSC), Social Competence (SC), Family Support (FS), Social Support (SS), and Structure (Str), ranging from minus three to two standard deviations below the mean to 1 to 2 standard deviations above the mean.

The MDISC and MDIFF parameters of the RESI-M items were different for each item, which supports the idea that all items contribute differentially to the measurement of the dimensions of resilience. This is not a problem for the measurement of the attribute because it is a realistic expression of the differential content of the items and the conceptual structure of the construct being measured. All items had high or very high discrimination, which indicates that they have the ability to distinguish with high accuracy between individuals who have low or high levels of the dimensions assessed; in the context of caregivers of cancer patients, this could be very useful to detect who would need specific psychological intervention and describe them with high accuracy in the RESI-M framework.

Regarding the precision of the estimates of the latent traits by means of the information functions of the five dimensions of the RESI-M, IRT modeling made it possible to detect within the RESI-M which of the factors and at which levels of the traits there was more measurement precision and therefore more reliability. The findings obtained indicate that the measurement precision had a bimodal form, in that the further away from the mean the subject’s position is, the higher the precision will be. This bimodal form of the information function suggests that the construct is sensitive to individual differences at the extremes of the construct but does not appear to be recommended for scores near the mean because of the greater measurement error at this level of the score. This seems unusual; however, it may be reasonable in the measurement of resilience, given that this construct emerges or is clearly observable when the subject is exposed to adverse factors, and the expression of resilient behavior may show consistency in these extreme situations. A practical implication is that because all factors had a decrease in informativeness around the mean, if one wanted to improve the scale in terms of greater coverage in the range of latent scores, creating items that are informative at average levels of resilience would be appropriate. One practical implication for the use of the instrument is that the description of the resilience attribute may be less appropriate for groups at the middle level of the RESI-M and more accurate and consistent at both ends of the construct. Overall, in the future, practitioners using the RESI-M in caregivers of oncology patients could reliably determine whether the caregiver has high or low levels of the dimensions assessed without additional analysis; this is consistent with what has been reported in previous research about IRT utility, level of reporting, and test-retest reliability (53).

Regarding the convergent and divergent validity of the RESI-M with the total scores of the BDI, BAI, WHOQoL-BREF, and ZBI, the latent scores in the factors of the instrument correlated with the scores of scales to measure depression (BDI), anxiety (BAI), quality of life (WHOQoL-BREF) and caregiver overload (ZBI); therefore, the hypotheses of association with variables were satisfactorily fulfilled. An important finding of the present study is that regardless of the number of items contained in each factor, the factors correlated congruently and statistically significantly with the scales. Therefore, the strongest correlations with the scores of the instruments were those of the Strength and Self-Confidence factor, which is the factor with the largest number of items; even the Structure factor, with only five items, correlated congruently and statistically significantly with the scales mentioned; therefore, we can conclude the validity of the estimates of the constructs that we obtained with IRT. The results of the correlations coincide with a previous study (27, 35) that evaluated the relationship between scores of the RESI-M factors, obtained with confirmatory factor analysis (CFA), and the total scores of depression and anxiety. In the present study, we also detected a negative association of RESI-M factors with depression and anxiety scores; however, we extended those findings by obtaining a positive correlation with the WHOQoL-BREF quality of life scores and a negative correlation with the ZBI scores. The theoretical congruence of the correlations and the correspondence with previous findings provide evidence for the validity of the RESI-M and its factor estimates in the IRT framework.

A limitation of the study is that objective measures of health were not used; therefore, future studies would benefit from establishing a relationship with measurements other than self-report, such as physiological measures or behavioral records. Another limitation refers to the non-probability sampling and the sample size of less than 1,000, which indicate that the estimates should be taken with due caution (although some simulation studies suggest that 500 participants may be adequate (54)), even within the population from which the sample was drawn (family caregivers of children undergoing cancer treatment at the Hospital Infantil de México Federico Gómez, National Institute of Health, in Mexico City).

## 5. Conclusion

The original five-dimensional structure of the RESI-M was confirmed. As a contrasting strategy, alternative structures were tested, specifically unidimensional and bifactor (one general dimension and five specific dimensions), but they were not strong enough to justify the use of a general score and interpret it theoretically. However, there are items with potential psychometric strength to create a possible general dimension, and future studies may confirm this psychometric property. The items of the subscales in general are shown to be representative of the measured dimensions and to contribute to the robust interpretation of their dimensions. The accuracy of the scores is high at the extremes, i.e., when the respondent scores below or above the mean. The overall reliability of the scores tends to be acceptable for group description and applied research purposes. Finally, the RESI-M scores show convergent validity in relation to the emotional responses of depression, anxiety and burden, as well as perceived quality of life.

Finally, we provide some suggestions for future lines of research. Due to the length of the instrument and imbalance in the content presentation of the subscales (number of items in each subscale), the moderate overall factor strength and the size of the interfactor correlations, an abbreviated version of the instrument could be developed. At the same time, in the present study, reliability by stability was not estimated, so it is suggested to estimate reliability at least at two different time points. Using a short-term and a long-term interval, the stability and dependability of the scores can be evaluated (55). Both aspects are conceptually different and provide different facets of score stability. The invariance or equivalence between groups was also not contrasted since the eligible samples were unbalanced; therefore, its evaluation (sex of the parents, sex of the oncology patient, etc.) is indicated from a non-proportional stratified sampling (with equiprobable or balanced strata). This type of contrast will help to establish the invariance of the estimated psychometric parameters or to describe differences.

## Data availability statement

The original contributions presented in this study are included in this article/supplementary material, further inquiries can be directed to the corresponding author.

## Ethics statement

This study is a part of the research project HIM/2015/017/SSA.1207 “Effects of mindfulness training on psychological distress and quality of life of the family caregiver”, which was approved by the Research, Ethics, and Biosafety Commissions of the Hospital Infantil de México Federico Gómez, National Institute of Health, in Mexico City. The patients/participants provided their written informed consent to participate in this study.

## Author contributions

JM, SJ, and FT-T: conceptualization. SJ, CM-S, and FT-T: methodology and writing—original draft preparation. SJ: software and formal analysis. RV-G and SJ: validation. CM-S and FT-T: research. FT-T and RV-G: resources. SJ and FT-T: data curation and project administration. FT-T, CM-S, and JM: writing—review and editing. SJ and CM-S: visualization. FT-T and JM: supervision and funding acquisition. All authors have read and agreed to the published version of the manuscript.
